# First indications that northern bottlenose whales are sensitive to behavioural disturbance from anthropogenic noise

**DOI:** 10.1098/rsos.140484

**Published:** 2015-06-03

**Authors:** P. J. O. Miller, P. H. Kvadsheim, F. P. A. Lam, P. L. Tyack, C. Curé, S. L. DeRuiter, L. Kleivane, L. D. Sivle, S. P. van IJsselmuide, F. Visser, P. J. Wensveen, A. M. von Benda-Beckmann, L. M. Martín López, T. Narazaki, S. K. Hooker

**Affiliations:** 1 Sea Mammal Research Unit, Scottish Oceans Institute, University of St Andrews, St Andrews, Fife KY16 8LB, UK; 2 Maritime Systems Division, Norwegian Defence Research Establishment (FFI), Horten 3191, Norway; 3 Acoustics and Sonar, Netherlands Organisation for Applied Scientific Research (TNO), PO Box 96864, 2509 JG The Hague, The Netherlands; 4 Acoustic Group, Centre for Expertise and Engineering on Risks, Urban and Country Planning, Environment and Mobility (CEREMA - DTer Est), F 67035 Strasbourg cedex2, France; 5 Centre for Research into Ecological and Environmental Modelling, University of St Andrews, St Andrews, Fife KY16 9LZ, UK; 6 Institute of Marine Research (IMR), PO Box 1870 Nordnes, Bergen 5817, Norway; 7 Kelp Marine Research, Loniusstraat 9, 1624 CJ Hoorn, The Netherlands; 8 Behavioural Biology Group, Leiden University, PO Box 9505, 2300 RA Leiden, The Netherlands

**Keywords:** bottlenose whale, anthropogenic noise, behavioural response, mitigation, naval sonar, *Hyperoodon ampullatus*

## Abstract

Although northern bottlenose whales were the most heavily hunted beaked whale, we have little information about this species in its remote habitat of the North Atlantic Ocean. Underwater anthropogenic noise and disruption of their natural habitat may be major threats, given the sensitivity of other beaked whales to such noise disturbance. We attached dataloggers to 13 northern bottlenose whales and compared their natural sounds and movements to those of one individual exposed to escalating levels of 1–2 kHz upsweep naval sonar signals. At a received sound pressure level (SPL) of 98 dB re 1 μPa, the whale turned to approach the sound source, but at a received SPL of 107 dB re 1 μPa, the whale began moving in an unusually straight course and then made a near 180° turn away from the source, and performed the longest and deepest dive (94 min, 2339 m) recorded for this species. Animal movement parameters differed significantly from baseline for more than 7 h until the tag fell off 33–36 km away. No clicks were emitted during the response period, indicating cessation of normal echolocation-based foraging. A sharp decline in both acoustic and visual detections of conspecifics after exposure suggests other whales in the area responded similarly. Though more data are needed, our results indicate high sensitivity of this species to acoustic disturbance, with consequent risk from marine industrialization and naval activity.

## 1. Introduction

Underwater noise generated by human activities such as shipping, exploration, naval sonar and other sources is considered by many international agencies to be marine pollution [1]. The extent to which noise pollution degrades the quality of marine habitats depends crucially upon the effects of noise on animals in the exposed environment. Concern for effects of underwater noise on cetaceans is marked because of their dependence on sound for communication, foraging and sensing the environment, and the tendency of some species (particularly beaked whales) to strand when exposed to intense sounds produced by naval sonars [2,3]. Spurred by these concerns, a number of recent studies have described and quantified changes in functional behaviour of free-ranging cetaceans to both simulated and real navy sonar signals [4–11]. The acoustic thresholds and types of behavioural responses vary across and within species [5,9], and also appear to be strongly affected by the context in which animals are exposed [6,12].

The species group most clearly associated with sonar-linked stranding events, beaked whales (Family: Ziphiidae), have been shown to be sensitive to simulated and real active sonar transmissions in key studies conducted near US Navy ranges: in the Bahamas Atlantic Undersea Test and Evaluation Center (AUTEC: Blainville's beaked whale, *Mesoplodon densirostris* [4,10]) and near the Southern California Offshore Range (SCORE: Cuvier's beaked whale, *Ziphius cavirostris* [7] and Baird's beaked whale, *Berardius bairdii* [11]). All five beaked whales exposed to sonar while tagged exhibited strong reactions, including cessation of foraging, avoidance and heightened swimming speed, that started at rather low received levels and persisted for several hours after the end of exposure periods [4,7]. The response of an individual *B. bairdii* was similar to that of the other two species, but was of shorter duration after sound transmission ceased [11]. Responses of individuals tagged with high-resolution DTAGs were consistent with larger scale trends in movement [4] and echolocation behaviour of whales during actual exercises on the AUTEC range [4,10]. These small but crucial datasets indicate that beaked whales are particularly sensitive to sonar, supporting the suggestion by Cox *et al.* [3] that behavioural responses may be an important factor leading to their stranding. Thus far, such studies have been carried out exclusively in the vicinity of navy underwater training ranges, and the high level of sonar activity in such areas is clearly an important contextual feature limiting our ability to predict responses of beaked whales in more pristine habitats.

The northern bottlenose whale, *Hyperoodon ampullatus*, is a beaked whale that ranges over high latitudes of the north Atlantic Ocean [13]. The current population of northern bottlenose whales is thought to be much-reduced due to intense historical whaling [14,15]. These whales were noted for their curiosity towards unusual sounds and their allegiance towards wounded companions, which, unusually among whaling vessels, led to harpoon guns mounted both fore and aft, and allowed whalers to capture entire groups [16,17]. Underwater noise, which is expected to increase in the species' habitat as human activities expand into deep Arctic waters [18], is a potential threat to the recovery of the species. One northern bottlenose whale stranded in 1988 on the island of Fuerteventura during a sonar exercise [19], but to date there have been no direct observations of how this species might respond to noise exposure. While many aspects of the diving and foraging behaviour of the northern bottlenose whale appear to be similar to those of other beaked whales [20–22], the observations of whalers that northern bottlenose whales have social defences against threats suggests that they might be less at risk of flight responses that could lead to stranding. Here, using a dose-escalation experiment, we tested how bottlenose whales in a habitat far from any naval testing range responded to naval sonar.

## 2. Material and methods

We conducted a controlled exposure of naval sonar to an aggregation of bottlenose whales in which one individual was tagged with a sound and movement-recording datalogger attached to the whale by suction cups (DTAG [23]). The protocol for sonar exposure specified that the sonar source was to start transmissions at a planned distance of approximately 1 km from the tagged whale after at least 8 h of baseline tag data were recorded. The tag was programmed to release after being attached to the whale for 18 h.

During the sonar exposure, the source vessel (RV HU Sverdrup II) followed a pre-determined course, independent of the movement of the tagged whale or other whales, while towing a sonar sound source at 90–100 m depth. The sonar transmission consisted of one hundred and four 1 s duration 1–2 kHz hyperbolic upsweep pulses at 20 s intervals. The source level of the sonar pulses increased by 1 dB per pulse from 152 to 214 dB re 1 μPa m over 20 min (61 pulses), and the remaining pulses were transmitted for 15 min at a source level of 214 dB re 1 μPa m.

Before, during and after the sonar exposure, observers on the source vessel recorded visual and acoustic detections of northern bottlenose whales. Whales were sighted using the naked eye and 15×80 mounted binoculars, and clicks were detected using a hydrophone array [24] towed at 90–100 m depth. All visual detections were classified as new sightings, unless they were recognized as having been sighted previously (based upon group location, size and natural markings), in which case they were classified as re-sightings. Group sizes and the number of whales within 200 m of the tagged whale group were recorded using the protocol specified in Visser *et al.* [25]. Acoustic detections were scored as presence or absence within 5 min intervals. Representative received levels of the sonar pulses near the experiment were computed using the BELLHOP propagation model [26], including a range-dependent bathymetry and sound speed profile measured prior to the exposure experiment. Range- and depth-dependent incoherent transmission loss was computed, using the location of the source at the start of the exposure experiment. The transmission loss was computed in 360° around the source using steps of 2°. The source was modelled as a point source, with a source depth of 97 m (based on the value of the depth sensor on the source), an opening angle of ±60° to account for the source directionality, and a source level of 214 dB re 1 μPa m (the maximum RMS source level during the experiment). We ran BELLHOP at the centre frequency of 1500 Hz, using 500 rays in order to achieve a convergence of the computed transmission loss.

Data from the tag were converted to pressure, acceleration, magnetic field strength, and pitch, roll and heading in the whale-frame axis using standard methods [23]. The calibrated received sound pressure level (SPL) was measured over a 200 ms RMS averaging window for each sonar ping [5]. This received SPL was calculated by summation of the acoustic power over the 1–40 kHz third-octave bands that had a signal-to-noise level more than 10 dB. The position of the tagged whale at the start of the experimental sonar transmissions was geo-referenced by finding the position with the smallest RMS difference between observed sonar arrival times and predicted arrival times based upon the dead-reckoned track. The time-synchronization between the tag and the GPS-synched sonar transmission clock was accurate to less than ±0.5 s, so we are confident of our whale track locations to approximately ±0.75 km during the sonar transmission period.

**Figure 1.**
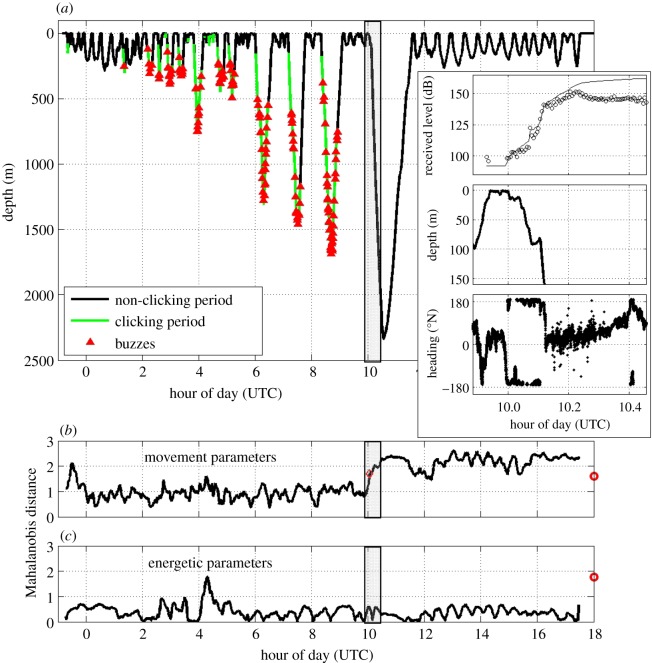
(*a*) Time-depth record of tagged whale ha13_176a with the timing of the sonar exposure period lightly shaded between vertical lines. Sounds are marked by colour: black indicates periods when no foraging sounds were produced by the tagged whale, green shows periods when the tagged whale was producing foraging echolocation clicks and red triangles indicate buzzes (i.e. likely foraging attempts). The inset box highlights data during the sonar exposure period and shows received levels of the sonar with ping by ping SPL (in dB re 1 μPa) shown as ‘open circles’ and cumulative sound exposure level (in dB re 1 μPa^2^ s) as a solid line (top), zoomed whale depth truncated at 150 m to show detail at the start of the dive (middle) and whale heading (bottom). (*b*) Mahalanobis distance values for movement parameters. Red circle on the right indicates the response threshold and red diamond in the graph indicates when the response threshold was exceeded. (*c*) Mahalanobis distance values for energetic parameters. Red circle on the right indicates the response threshold.

Audio files recovered from the experiment and baseline tags were examined aurally, and spectrograms were inspected visually to identify the start and stop times of foraging sounds (foraging echolocation clicks and buzzes, which are likely to represent prey-capture attempts) produced by the tagged whale (figure 1*a*), as well as those produced by other whales [21]. Sounds were ascribed to the tagged whale or not based upon their intensity in the tag recording and the timing of arrival of the sounds on stereo tags (for baseline records only). Flow noise (less than 500 Hz) recorded on the audio channel was correlated with speed through the water [27] measured during steep (more than 60° pitch) transit periods to estimate speed throughout the tag record (except for depths less than 10 m, where 2 and 3 m s^−1^ were each modelled to bracket expected speeds near the surface). Estimated speed was combined with pitch and heading data to estimate a dead-reckoned track of the whale [23,28]. Sonar arrival times were marked in the audio record by cross-correlation with the transmitted waveform.

Behaviour of the tagged whale during the pre-exposure baseline period was summarized using two sets of quantitative variables calculated at a common sampling rate of 5 Hz. The first set of variables, movement parameters, was designed to detect predicted movements during avoidance and consisted of dive profile wiggliness (proportion zero crossings in the first difference of the depth time series), whale heading (decomposed into sine and cosine components) and variability of animal pitch and heading. The second set, energetic parameters, was designed to track locomotion effort of the whale and consisted of overall dynamic body acceleration (ODBA [29]) and pitching movements relative to the body axis. As these energetic parameters can be sensitive to tag position on the animal, we confirmed that tag movements on the body did not influence the outcome of this analysis by inspection of the data to confirm that overall ODBA values were consistent over the time period of the tag deployment. The first dive after tagging was not included in the baseline period to reduce possible short-term influences of the tagging procedure. For both sets of variables, we calculated the Mahalanobis distance between the baseline-period average value and the averages of 15 min windows centred at 1 min intervals [11]. We set a threshold for change-point detection at the 95th percentile of expected Mahalanobis distance. This threshold was derived from resampling (from baseline) 100 000 periods of the same duration as the 35 min exposure, and setting the response threshold at the 95th percentile of the maxima of the resampled periods (figure 1*b*,*c*).

To compare dives of the exposed whale with those of other tagged northern bottlenose whales, we analysed data from eight other tag records obtained from the Gully, eastern Canada, and five other tag records from Jan Mayen. All of the Jan Mayen records and two of the Gully records used DTAGs. Four Gully records used Little Leonardo 3MPD3GT loggers [30] and two were time-depth recorder data previously reported by Hooker & Baird [20]. All dives greater than 10 m depth were extracted from time-depth records. Dives were segmented into three phases: descent (from the surface until first excursion greater than 85% of maximum depth), ascent (from the final excursion greater than 85% of maximum depth to surfacing) and bottom (from end of descent to start of ascent); we also measured the surface interval after each dive (from surfacing until the start of the next dive) [31]. For each dive across all tag types, basic dive parameters were calculated, including: maximum depth, dive duration, descent rate, ascent rate, bottom duration and surface interval [31]. For tag types including acceleration sensors (DTAGs and 3MPD3GT), kinematic dive parameters included ascent pitch, descent pitch, variability of heading, variability of pitch and ODBA. ODBA values were normalized by the whale-specific median value to help account for the effects of tag locations on the animals. Dives were clustered into short-shallow or long-deep dive types by *k*-means clustering based upon dive duration, maximum depth, and descent and ascent rates. For basic dive parameters, all dives by all tagged whales were included in the analysis: 477 shallow dives and 79 deep dives. For the kinematic dive parameters, only data from whales tagged with DTAGs and 3MPD3GTs were included: 379 shallow dives and 57 deep dives. The Mahalanobis distance between each dive and the dive-type-specific mean value were calculated for both basic and kinematic dive parameters. We also estimated the probability of observing a Mahalanobis distance as extreme as that of the deep exposure dive (under the null hypothesis that distances were normally distributed and not dependent on exposure).

## 3. Results

We surveyed the waters near Jan Mayen from 22 June to 10 July 2013. During that period, 220 groups of *Hyperoodon* were sighted, with group sizes ranging from 1 to 10 animals, with a mean (s.d.) group size of 3.5 (1.7) individuals. On 25 June, we tagged an individual (ha13_176a) within a group of six animals that approached the tag boat. The tagged whale was later seen in a group of three to four animals. During the 10.5 h pre-exposure period, the tagged whale exhibited a diverse set of shallow and deep dives. Clicking and buzzing foraging sounds at amplitudes indicating they were most likely produced by the tagged whale were recorded, primarily during the deeper dives (figure 1). A large number of fainter foraging sounds, judged to be produced by other whales, were also recorded.

**Figure 2.**
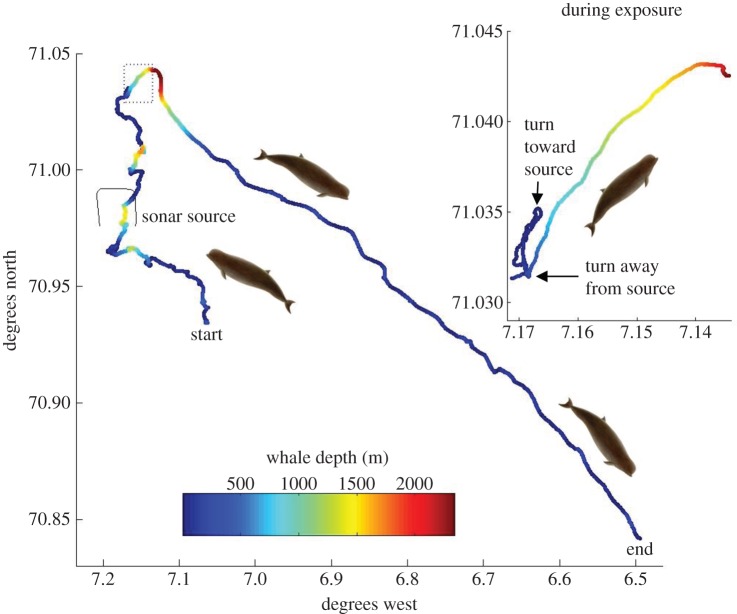
Geometry of the controlled exposure experiment and movement of the tagged whale before, during and after the sonar exposure. Colour of the track indicates whale depth (m). The track of the sonar source during the exposure is plotted as a thin black line. The source moved counterclockwise during the exposure. Inset box shows detail of whale movement during the 35 min exposure period, indicated by a dashed box on the overall track.

The sonar exposure started when the tagged whale had been on a dive to 100 m for a duration of 7.8 min (figure 1, inset). Based upon the geo-referenced track, the whale was approximately 5 km away, almost due north of the source when sonar transmissions started (figure 2). The whale returned to the surface for 3.6 min, taking 15 breaths. At the start of the next dive, the whale had turned towards the source with SPL of the sonar at a received level of 98 dB re 1 μPa (figure 1*a*, inset box top), which was, according to our criteria, at least 10 dB above the noise level in at least one 1/3-octave band between 1 and 2 kHz. Noise recorded by the DTAG can be affected by flow noise, resulting in elevation of the noise levels above ambient noise. However, the lowest 1/3-octave noise levels within the sonar band were comparable with those predicted by the Wenz curves [32] for deep sea ambient noise levels (approx. 90 dB re 1 μPa) for a Sea State=3 (representative of the weather conditions during the exposure experiment), although some increase in ambient noise level is expected when the animal was closer to the surface.

The received SPL continued to increase as the whale made an unusually smooth descent to approximately 90 m. The time-series Mahalanobis distance analysis for movement detected the onset of a behavioural response as the whale began swimming along an unusually straight course as it passed 60 m depth, still approaching the source, when received SPL of the sonar was at 107 dB re 1 μPa (figure 1). Next, the whale made a sharp change to greater depth turning away from the source as the received SPL of the sonar was 130 dB re 1 μPa.

The whale continued to dive to deeper depth throughout the sonar exposure, with full source-level transmissions of 214 dB re 1 μPa m and the maximum received SPL of 151 dB re 1 μPa occurring as the animal reached 789 m depth. Received levels decreased slightly as the whale dove more deeply, moving away from the sonar. Sonar transmissions stopped when the whale was at 2074 m depth, with a cumulative sound exposure level received by the whale of 162 dB re 1 μPa^2^ s. After reaching a maximum depth of 2339 m approximately 10 min after the sonar stopped, the whale turned towards the surface where it re-surfaced an estimated 5.4 km from the location where it had started the dive. No foraging clicks were detected from the tagged whale or other nearby animals during the long and deep dive recorded during the exposure period. In the post-exposure period, the whale continued to show unusually directional horizontal movement (figure 2) during a series of stereotyped shallow dives (figure 1*a*). This unusual movement behaviour continued until the tag detached from the whale 7.1 h after the end of the sonar exposure, roughly 30–36 km from its location when the exposure started (figure 1*b*). The Mahalanobis statistic value for energetic parameters did not show unusual values during the exposure period, relative to values recorded during the pre-exposure baseline period (figure 1*c*).

**Figure 3.**
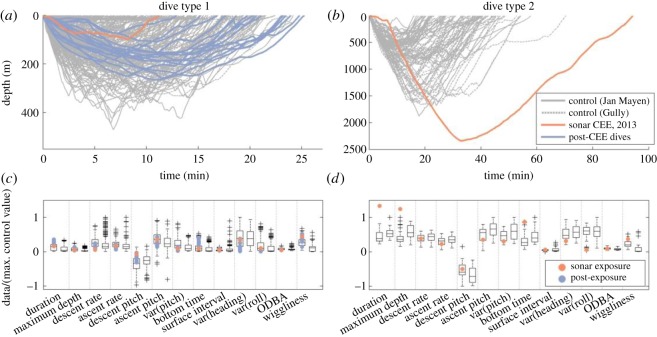
Dive profiles and dive parameters by dive type. The figure includes all 477 shallow-short dives (*a*,*c*) and 79 long-deep dives (*b*,*d*) recorded from 14 tag records of northern bottlenose whales (eight records from the Gully, six from Jan Mayen). Note unequal *x*- and *y*-axis scales (*a*,*b*). Dives without sound exposure are plotted in grey; dives overlapping controlled exposures to 1–2 kHz sonar in orange; and post-exposure dives by the exposed whale in blue. Paired box-plots are shown for each dive parameter of each dive type (*c*,*d*), with data from Jan Mayen (including the exposure whale) to the left and data from Gully to the right for each parameter. For the box-plots, to facilitate showing many variables on a single plot, all values were scaled before plotting by dividing by the maximum of the absolute value of all control observations. Here, ‘control’ observations include dives by unexposed animals, as well as pre-exposure dives by the exposed whale in Jan Mayen. Black boxes span 25–75th percentiles, black horizontal lines mark medians, error bars span 1.5 interquartile ranges and+symbols indicate more extreme values. Dive parameters from exposure and post-exposure dives are plotted individually. Symbol and colour-coding match (*a*,*b*).

**Figure 4.**
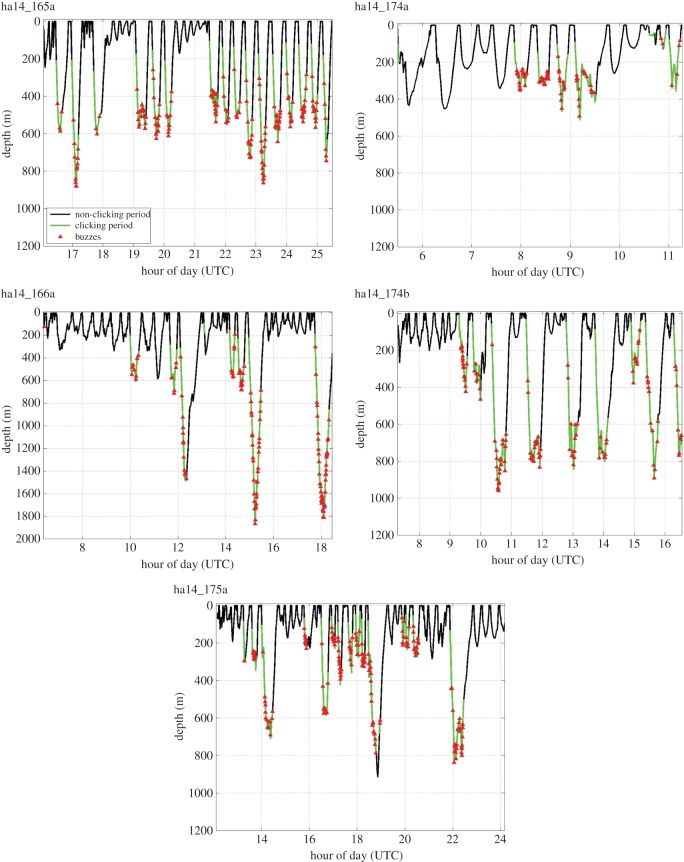
Foraging sound production relative to diving depth in five DTAG control deployments recorded in Jan Mayen in summer 2014. Note that foraging sounds (clicks and buzzes) tended to be produced during deeper dives conducted by most individuals. Note unequal *x*- and *y*-axis scales on different panels.

In comparison to baseline data from this and other whales (table 1), the dive conducted by the exposed whale was the longest and deepest dive (94 min, 2339 m) recorded for the species (figures 3 and 4). This dive had strikingly unusual basic dive parameters (Mahalanobis distance analysis, *p*=0.002) relative to all other deep dives recorded (*n*=79), with the strongest differences in parameters related to dive depth and duration (figure 3*b*,*d*). Some kinematic parameters of the long and deep exposure dive deviated from baseline values (in particular, variation in roll and heading and wiggliness of the dive profile). Overall, however, the kinematic Mahalanobis distance for the exposure period fell within the expected range for baseline dives (*p*>0.05). The shallow exposure dive and post-exposure dives were not so different from control shallow dives, although some features of some dives were potential outliers relative to control dives (figure 3*a*,*c*).

**Table 1.** Details of all tag deployments.

deployment ID	location	date	duration (h)	tag type
ha13_176a	Jan Mayen	25 June 2013	18.2	DTAG
ha14_165a	Jan Mayen	14 June 2014	9.4	DTAG
ha14_166a	Jan Mayen	15 June 2014	12.3	DTAG
ha14_174a	Jan Mayen	23 June 2014	5.8	DTAG
ha14_174b	Jan Mayen	23 June 2014	12.2	DTAG
ha14_175a	Jan Mayen	24 June 2014	12.0	DTAG
ha_01	Gully	9 July 1997	2.5	TDR
ha_02	Gully	24 Aug 1997	27.9	TDR
ha07_218a	Gully	6 Aug 2007	7.1	DTAG
ha13_248a	Gully	5 Sept 2013	2.6	DTAG
3M1	Gully	7 Aug 2011	8.8	3MPD3GT
3M2	Gully	8 Aug 2011	2.4	3MPD3GT
3M3	Gully	11 Aug 2011	7.6	3MPD3GT
3M4	Gully	6 Sept 2013	19.9	3MPD3GT

**Figure 5.**
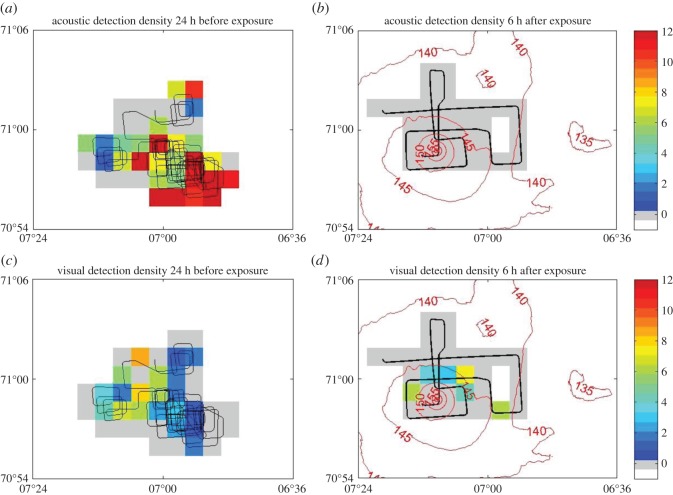
Both acoustic (*a*,*b*) and visual (*c*,*d*) detections of northern bottlenose whales decreased in density comparing rate of detection during 24 h prior to sonar exposure (*a*,*c*) and 6 h after sonar exposure (*b*,*d*). The track of the research vessel is shown (black line) together with coloured density of detections for each 2×2 km cell surveyed. Acoustic detections were quantified as number of 5 min intervals in which clicks were detected per hour. Visual detections consisted only of new groups identified and were quantified as average sightings per hour. Contour lines (*b*,*d*) indicate the SPLs (in dB re 1 μPa), averaged over 0–500 m, representative of shallow dives.

In terms of responses of other whales to this exposure, detection rates of northern bottlenose whales by both our acoustic and visual surveys dropped substantially in the 6 h period after the sonar exposure, relative to the number of acoustic and visual detections in the 24 h period prior to the exposure (figure 5). Visual sightings of groups of whales (not counting re-sightings) declined from 8.5 groups per hour in the 24 h before sonar transmissions to just 1.3 groups per hour after. In the 24 h before the start of exposure, acoustic detections were made in 58% of all 5 min periods, corresponding with the high number of visual sightings before the exposure started. No acoustic detections at all were made in the surveyed area after the sonar exposure (figure 5).

## 4. Discussion

The results of our experiment demonstrate that northern bottlenose whales may be highly sensitive to disturbance by underwater noise, with a scale of behavioural response at least as strong as that exhibited by other beaked whales [4,7]. The behavioural response of the tagged animal was typified by a strong change in swimming and diving behaviour starting at received levels (SPL) of 107 dB re 1 μPa, indicating that its movement was driven by response to the sonar signals received up to that point rather than part of natural functional behaviours. Deep dives before the sonar exposure had variable movements and were characterized by production of foraging clicks and buzzes, typical of the deep feeding behaviour of other tagged northern bottlenose whales in our baseline dataset from Jan Mayen (table 1 and figure 4) and other beaked whales [21]. The dive initiated by the whale during the sonar exposure appears to reflect a cryptic deep dive strategy to avoid a perceived threat, which is also similar to the responses of other exposed beaked whales, and may reflect adaptations to avoid predation by killer whales [33]. Avoidance movements and lack of sound production related to foraging effort continued for the 7.1 h that the tag remained attached after the exposure, indicating a prolonged response, and substantial movement at least 30–36 km away from its position before the sonar started.

The initial movement of the tagged whale towards the sound source prior to the observed response at higher received SPL (figure 2) was reminiscent of whalers' descriptions of enticing animals by sound [16]. The initial approach behaviour (initiated at 98 dB re 1 μPa received level) followed by a strong avoidance response (initiated at 130 dB re 1 μPa) suggests that the sound which was attractive at lower received levels became aversive after it escalated to higher received levels. An initial approach to sound sources would lead to higher sound exposure levels, but whether or not this also leads to increased severity of disturbance is unclear.

A strong decline in visual and acoustic detections of bottlenose whales after the sonar exposure (figure 5) further indicated that many animals in the proximity of the sonar were affected by the exposure. The lack of any acoustic detections after the exposure indicates that animals moved away from the area and/or stopped producing foraging sounds and the strong decline in visual sightings is consistent with more cryptic surfacings and/or wide-scale avoidance movements away from the sonar. Acoustic modelling ground-truthed by our measurements of the sound field indicated that the area we surveyed after the sonar exposure was predicted to have had cumulative sound exposure levels of at least 140 dB re 1 μPa^2^ s. While we cannot reconstruct the precise exposure received by other animals in the area or their response thresholds, the reduction in the numbers of animals in the area and cessation of echolocation-based foraging behaviour are fully consistent with the clearly documented response of the tagged whale. We would assign a severity score of 8 (on our defined severity scale from 1 to 9 [5,34]) due to the large-scale and sustained nature of the avoidance and cessation of feeding. As our tagging and observation systems were not capable of measuring the full duration and spatial extent of the response, the duration and spatial scales reported here should be considered minimum estimates. There remains uncertainty as to the full scale of habitat degradation caused by the sonar exposure. Longer term observations of animal residency within the Jan Mayen habitat would be useful to determine the natural degree of fluctuations in whale numbers, and to better document how habitat might be affected by noise.

While we must remain cautious in extrapolating results from a single experiment to northern bottlenose whales generally, the strong responses observed in our study are consistent with typically sustained and large-scale responses of other beaked whales to sonar exposure [4,7,10]. Cuvier's beaked whales are the predominant species recorded in sonar-related strandings [2], but their more temperate and near shore distribution is closer to populated beaches where they would be found after stranding. Only one northern bottlenose whale has been reported to strand in association with naval sonar [19], but this animal was extralimital. It is therefore difficult to compare the stranding rates of bottlenose whales to those of Cuvier's because their core habitat is usually far from populated beaches [14].

In terms of comparing fine-scale behavioural responses with those observed for other beaked whale species, one *Z. cavirostris* may have switched from a putative shallow dive to a deep dive during sonar exposures at received levels of 89–127 dB re 1 μPa [7], similar to what appears to have taken place for the deep dive in our study (figure 3). However, our study animal did not increase locomotion effort as part of its avoidance response, as was observed in the other experiments with beaked whales [7]. Using a similar time-series-based Mahalanobis distance metric, the single *B. bairdii* had a much shorter response duration than we observed for the bottlenose whale [11]. Previous studies of *M. densirostris* [4], *Z. cavirostris* [7] and *B. bairdii* [11] have occurred in areas of frequent military sonar activity. Overall, we found similar characteristics of the type of response by a beaked whale in the more pristine habitat of our study area, which is far from locations with regular naval sonar activity. However, we lack information on the ranging behaviour of this population of whales, so it is that possible that individuals have experienced sonar signals elsewhere.

Based upon the preliminary information provided by our study, northern bottlenose whales should be considered to be sensitive to acoustic disturbance, as are other beaked whale species. There are legislation and policy developments requiring prevention of deleterious effects of anthropogenic noise. In the USA, NOAA is developing an Ocean Noise Strategy to address underwater noise impacts on the environment. Underwater noise is formally defined as pollution in article 3 of the European Union's Marine Strategy Framework Directive (2008/56/EC), that aims to achieve Good Environmental Status (GES) of the marine environment by 2020. The Marine Strategy Framework Directive includes 11 descriptors of GES, one of which is introduction of energy, including underwater noise. We recommend that more studies of how members of this species respond to noise sources in their environment should be collected. The potential for habitat degradation due to ‘impulsive’ sound (including sonar, explosions, air guns and pile-driving) is recognized to be of special concern [35]. Much of the temperate habitat of northern bottlenose whales is already impacted by anthropogenic activity [14]. The anticipated increase of human activities and associated noise emissions in the Arctic ocean [18,36] will increase the acoustic exposure in previously pristine areas. Overall, our results indicating high sensitivity of this species raise concerns about degradation of the quality of northern bottlenose whale habitat.

## Data accessibility

Supporting data are available from dryad.org.
